# Acute Pancreatitis Associated with Amoebic Liver Abscess

**DOI:** 10.1155/2013/717393

**Published:** 2013-04-02

**Authors:** Jayant Kumar Ghosh, Vinod Kumar Dixit, Sangey Chopel Lamtha, Sundeep Kumar Goyal, Pankaj Kaushik

**Affiliations:** Department of Gastroenterology, Institute of Medical Sciences, Banaras Hindu University, Varanasi, Uttar Pradesh 221005, India

## Abstract

We present a rare case of acute pancreatitis in a 50-year-old man with amoebic liver abscess. He had a right lobe liver abscess along with markedly elevated serum lipase and amylase levels and edematous pancreas. Liver abscess was aspirated. The patient was managed conservatively with antibiotics and improved without any complications. Acute pancreatitis associated with ALA is not reported in the literature till date.

## 1. Case Report

**Figure 1:**
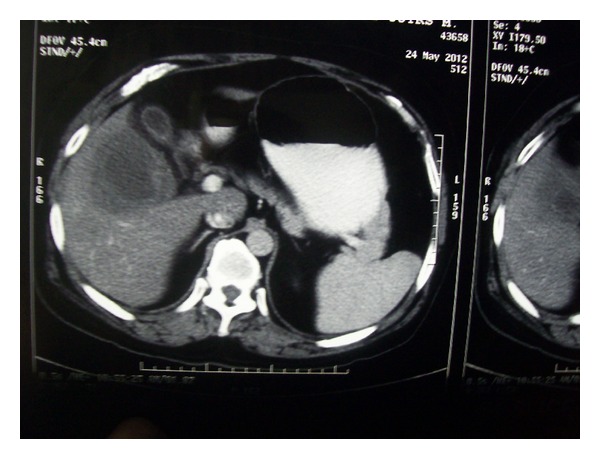
CECT abdomen showing right lobe liver abscess and edematous pancreas.

A 50-year-old male, nonalcoholic, presented with pain in right upper abdomen for the last 7 days which had increased in severity in the last 24 hours. He had not passed flatus for the last 12 hours. The patient had a history of acute diarrhea 1 month back. At admission patient was conscious, febrile and had diffuse upper abdominal pain which was severe in intensity. Abdomen was distended and bowel sounds were absent. There was tender hepatomegaly. Spleen was not palpable. No free fluid was detected clinically. There was no past history of diabetes, hypertension, and abdominal/biliary surgery. His investigations showed leukocytosis (total leukocyte count = 18,000/mm^3^). Serum lipase and amylase were markedly elevated (1788 mg/dL and 1365 mg/dL, resp.). X-ray abdomen showed distended bowel loops. Ultrasonography (USG) of abdomen was done which revealed an abscess cavity of 8 × 8 × 7 cm^3^ in the right lobe of liver situated near the surface of the liver. Serum IgG *Entamoeba histolytica* was positive. No gall bladder or common bile duct stones were seen in the USG. He had mild hypocalcaemia (serum calcium level = 8.2 mg/dL). Serum lipid profile, glucose, liver function tests, renal function tests, and thyroid profile were within normal limit. X-ray chest was unremarkable except for prominent bronchovascular markings. Arterial blood gas analysis was almost normal except for low calcium level. On day 2 of hospital admission, contrast enhanced CT (CECT) scan of abdomen was done which showed a large right lobe liver abscess associated with edematous pancreas without any necrosis or acute fluid collections (Figure 1). The modified CT severity index (CTSI) was 4/10. No fistulous communication between liver and pancreas or other organs could be demonstrated in the CECT abdomen. Patient was managed with intravenous fluid, intravenous antibiotics, that is, metronidazole and meropenem. Liver abscess was aspirated under USG guidance and about 250 mL of anchovy sauce pus was aspirated. Gram stain and culture of the pus were negative. The pus was also examined for pancreatic enzymes which were within normal limits. On day 2 of hospitalization patient had normal bowel sounds. Gradually oral feeds were built up. USG was repeated on day 7 of hospital admission which showed a resolving abscess cavity of 6 × 5 × 5.5 cm^3^ and upon aspiration no pus came out. The patient showed gradual improvement and was discharged on day 10 of hospitalization. Patient was reviewed after 15 days and there were no complications noted; however, USG abdomen still showed a hypoechoic area in the right lobe of liver about 4 × 3 × 3 cm^3^ suggestive of resolving abscess cavity.

## 2. Discussion

Pyogenic liver abscesses develop via seeding through portal circulation, directly via spread from biliary infections or from surgical or penetrating wounds and also from systemic organs via haematogenous spread. But in our case it was an amoebic liver abscess associated with acute pancreatitis. There was a recent history of acute diarrhea which might have led to amebic liver abscess. Anatomically, at least in this case it is difficult to explain that pancreas and liver might had involved from a common source. Most probably two diseases might have occurred simultaneously. Pyogenic liver abscess associated with acute pancreatitis was first reported in 1945 by Shallow et al. [1], which was detected perioperatively. Pyogenic liver abscess in chronic alcoholic pancreatitis that was reported by Gundling et al. in 2004 was due to a pancreaticohepatic fistula [2]. However, in our case no fistula could be documented. Pyogenic liver abscess complicating biliary stricture due to chronic pancreatitis has been described in the literature [3]. Similarly, pyogenic hepatic abscess as a complication of sump syndrome was also reported in 2000 by Hiura et al. [4].

## 3. Conclusion

Pyogenic liver abscess in association with acute or chronic pancreatitis or with biliary tract diseases is a well-known entity. However, amoebic liver abscess in association with acute pancreatitis was never described in the literature. Probable common route of infection through the portal circulation which got infected from amebiasis in the colon does not make anatomic sense. It is most likely possible those two diseases might have occurred simultaneously.
